# What problems make students struggle during their undergraduate medical education? A qualitative exploratory study

**DOI:** 10.12669/pjms.36.5.2267

**Published:** 2020

**Authors:** Anbreen Aziz, Usman Mahboob, Ahsan Sethi

**Affiliations:** 1 Dr. Anbreen Aziz, BDS, MHPE. Department of Medical Education, HBS Dental College, Islamabad, Pakistan; 2 Dr. Usman Mahboob, MBBS, MPH, FHEA, DHPE, Fellow FAIMER. Assistant Professor in Medical Education. Institute of Health Professions Education & Research (IHPER), Khyber Medical University, Peshawar, Pakistan; 3 Dr. Ahsan Sethi, BDS, MPH, MMEd, FHEA, MAcadMEd, FDTFEd, PhD. Assistant Professor and MHPE/PhD Supervisor, Institute of Health Professions Education & Research (IHPER), Khyber Medical University, Peshawar, Pakistan

**Keywords:** Problems, Struggling Medical Student, Psychological distress, Impact

## Abstract

**Objective:**

To explore problems faced by struggling undergraduate medical students and their impact on student’s academics.

**Methods:**

A qualitative case study was carried out from March to August 2019. Semi-structured interviews were conducted with a purposive sample of sixteen struggling students. The interview questions were validated and then piloted to ensure clarity. All interviews were audio recorded and transcribed verbatim. Due to sensitive nature of the data, confidentiality and anonymity was ensured. Thematic analysis was employed to get meaning with in the data set. All authors ensured analytical triangulation by independently analyzing the data before developing consensus on the codes and themes.

**Results:**

Eleven sub-themes under four major themes emerged from the transcripts. Participants were found to be struggling with emotional, academics and family related problems. Psychological distress was caused by factors such as fight with friends, one-sided love, and hard financial times. Time management, lack of attention and commitments at home were few of the other problems. Problems had an impact on students as they were demotivated, lost focus in their studies, could not perform good and few participants even failed in examinations.

**Conclusions:**

Students were struggling more with emotional and family related problems and less with academics related problems. They were impacting student’s academics. The study site had a robust mentoring program however, struggling students need timely identification and more dedicated time to help them manage their problems. Stress relieving activities and counselling sessions may frequently be added, and academically underachieved students should also be given extra care, support, and guidance.

## INTRODUCTION

Medical schools are responsible for ensuring that graduates are knowledgeable, skillful and professional to meet society’s expectations.^1^ Numerous studies of past have shown high prevalence of self-reported psychological distress among medical students as compared with the general population.^2^,^3^ Although optimal level of stress is good for students during academic assessments, psychological distress may affect academic performance.^2^ The term psychological distress describe condition of a person who experiences emotional suffering such as feelings of stress, anxiety, depressed mood, and burn-out.^2^ Various factors such as personality, life events, responsibilities, medical school workload, curriculum and/or exposure to patient death may lead to psychological distress.^4^ Moreover, the climate of medical education can be stressed, with impact on academic performance and health.^5^ The students while going through undergraduate training may struggle with personal or academic problems and are termed as struggling students.^6^

Past studies assessed the sources and prevalence of stress and burnout in all medical students via pre-validated questionnaires such as *Copenhagen Burnout Inventory (CBI),^7^ Depression Anxiety Stress Scales (DASS-21)^8^* and *Kessler10 Psychological Distress (K10).^9^* In contrast, our study qualitatively explores the problems faced by the struggling students identified by the institutional mentoring program. The purpose of the study was to help strengthen mentoring program, by collecting in-depth data regarding problems and their impact on student’s academics.

## METHODS

A qualitative case study was carried out over a period of six months (March-August 2019). Ethical approval was granted by Ethics Review Committee (Riphah/IIMC/ERC/18/0313, Dated: February 11, 2019), Islamic International Medical College, Rawalpindi.

### Participants

The sampling frame included all the struggling students. Struggling students are those who face personal or academic problems during their undergraduate medical training.^8^ A purposive sample of sixteen struggling students was selected.

### Interview guide and data collection

An interview guide was developed. Open-ended questions were asked regarding problems participants were facing along with impact of those problems on their academics. The interview questions were validated by five medical education experts and then piloted with three students to ensure clarity. The participants were informed about the voluntary nature of participation. Informed consent was taken, and the data were collected over few weeks. All interviews were audio recorded and transcribed verbatim. Due to sensitive nature of the data, confidentiality and anonymity was ensured. Only the first author had access to the recordings. The transcripts were anonymized before these were shared with other authors for data analysis. All data were kept in a password protected Laptop.

### Data Analysis

Thematic analysis was employed to examine patterns within the data and to generate themes. Initially the authors familiarized themselves with the data by reading each line and segment carefully. In the next step, in-vivo codes were made to get rich descriptions of the data set. The codes merged to form categories and themes. All three authors analyzed the data independently, the codes and themes were discussed and agreed upon to ensure analytical triangulation.

## RESULTS

Participants were mainly from clinical years (62.5%) and majority were struggling females (62.5%) (Table-I). Our study has explored the problems of struggling students and impact of those problems on their academics. Struggling students were identified by the institutional mentoring program. Mentoring program with its fortnightly formal mentoring sessions is responsible to manage problems of students and to provide psychologist for counselling sessions within institute.

**Table-I:** Characteristics of the study participants.

Characteristics		Frequency	Percentage (%)
Gender	Male	6	37.5
	Female	10	62.5
Age (Years)	20-22	10	62.5
	23-25	6	37.5
Year of study	1^st^ Year	0	0
	2^nd^ Year	3 (1M, 2F)	18.75
	3^rd^ Year	3(F)	18.75
	4^th^ Year	5 (3M, 2F)	31.25
	5^th^ Year	5 (2M,3F)	31.25

*M-Male, F-Female.

Four themes along with eleven sub-themes were identified from data analysis (Table-II). Participants reported more emotional and family related and less academics related problems.

**Table-II:** Problems of struggling undergraduate medical students with their impact on academics.

Themes	Sub-themes	Representative Quotes
Emotional problems/ Psychological distress	Fight with friends	I am quite depressed these days due to fight with friends…they blamed me of not sharing study notes with them…thinking too much…started taking sleeping pills… unable to focus on studies (MaleY4#13)
	One-sided love	I love one of my class fellows…it is one-sided…I remain depress and irritable all the time…started smoking 15-20 cigarettes/day. Now my studies are being affected … failed few subjects (MaleY5#16)
	Financial crisis	I am stressed out due to financial crisis after a big loss in my father’s business…all savings and investments are gone…unable to concentrate on studies due to continuous stress of fee submission (FemaleY4#9)
Impact: Loss of focus in studies and failure in examinations		
Academics related problems	Time management	Supply is a shock for me. Our curriculum is integrated…lot of course and ongoing frequent assessments…Unable to manage time (MaleY4#14)
		My problem is of time management since 1^st^ year…I am a day scholar…when you are staying at home you are duty bound. Therefore, I am unable to focus on my studies and could not perform good in examinations (MaleY4#12)
Impact: Failure, loss of focus and poor performance in examinations		
Family related problems	Distractions	My study started effecting due to distracting factors and commitments…staying at paternal uncle’s home…cannot focus on studies…frequency of guest’s arrival is increasing day by day (FemaleY3#2)
	Commitments	I do not feel good… my mother is suffering from cancer…have to give time to mother and household…I am the eldest among siblings. This year, I could not perform good in examinations…how to study and get out of this emotional crisis (FemaleY2#3)
	Lack of attention	Situation of my home is very tense. My brother is schizophrenic…under treatment. My parents just pay attention to brother…I am not motivated to study…I failed an examination this year…used to be a good student in past (FemleY3#10)
Impact: Loss of focus, poor performance and demotivation		

*M-Male, Y-Year of study.

### Emotional problems

*Psychological distress* was found as the most common problem among the participants which was caused by factors such as *fight with friends*, *one-sided love* and *financial crisis*. One participant was depressed and lost focus in his studies due to fight with his old trustworthy friends. They blamed him of hiding study notes from them. A participant while explaining his one-sided love told that he even failed few subjects in examinations. Another participant shared that huge loss in her father’s business kept her under continuous stress of academic fee submission therefore, she lost focus in studies.

### Academics related problems

Struggling students had *time management issues* while staying at home and due to integrated curriculum in the institute. One of the participants told that he was a day scholar since the first year and was duty bound at home as he had to fulfill house chores. Whereas, a fourth-year student was in shock of first time failing a subject because he was unable to cope with the course content and frequent assessments.

### Family related problems

While staying at paternal uncle’s home, one struggling student was unable to focus on her studies due to *distractions* by frequent guests’ arrival. Whereas, another student had *commitments* at home being the eldest sibling as she was taking care of her ill mother. They lost focus in studies and performed poorly in examinations. Another participant was *lacking attention by parents* as all attention was grabbed by her ill brother therefore, she was not motivated to study and even failed in the examinations.

### Impact on academics

The students were demotivated, lost focus in studies, performed poorly, and failed in examinations due to various emotional, academics and family related problems.

## DISCUSSION

The study highlights the problems of struggling undergraduate medical students and its impact on their academics. Comparing with previous studies,^2^,^3^,^7^ our findings regarding problems are more or less same with few differences. In our study, psychological distress and family related problems were the most common problems faced by struggling students due to factors such as *fight with friends, one-sided love, financial crisis, distractions, commitments, and lack of attention*. While in a previous study, increased workload and highly demanding nature of medical studies were causing stress among students.^10^

Psychological distress includes stress, anxiety, depression and burnout in medical students that may be caused by personal factors, or the factors related to medical school training.^7^ In a previous study, overall causes of stress were academic factors such as long, academically rigorous, and emotionally taxing phases of undergraduate medical training period,^11^ while the emotional factors were the cause of stress in the first year MBBS.^12^ In our study psychological distress was caused by only emotional factors in fourth and fifth year students.

More than 300 million peoples of all ages suffer from depression globally and untreated depression may lead to suicide among university students.^13^ In a previous study, high stress level and low social support were the factors causing increased symptoms of depression in medical students.^14^ Moreover, financial difficulties were the second major stressor in medical students in a previous study.^15^ Similarly, in our study, financial crisis was the cause of emotional problems among struggling students. On contrary, a previous study reported that stressful environment of medical school caused emotional disorders and students felt sad and tired in response to life events which affected their academic performance.^16^ In our study, struggling students were losing focus in studies and their emotional problems were cause of their failure in examinations.

Medical education being the toughest and vast field, calls for proper *time management* to achieve the set goals. A participant shared that he could not manage frequent assessments within integrated curriculum. His time management issues led to first-time failure which was shocking for him. Another participant was unable to focus on studies due to house chores as he was a day scholar, therefore, could not perform good. There are certain tips provided in the literature that emphasize on the need to develop skills such as time and life management to sustain lifelong career in medicine.^17^

One of the participants was not motivated to study due to family related problem. Her parents were only paying attention to her schizophrenic brother. Though she was a good student in past, she failed the current year’s examination. In health professions education, motivation plays a significant role in the student’s academic performance as it is based on self-determination theory.^18^ According to the theory, motivation is multi-dimensional construct depending upon self-determined and controlled behaviors in different environments.^19^ Moreover, Internal regulation (intrinsic motivation) drives to pursue activity and is the most self-determined form of behavior which urges one to learn. In educational context, motivation is influenced by social experiences such as teacher’s support, extent of responsibility and early patient contact. Motivation can be enhanced by changing the educational environment and by supporting self-determined forms of motivation.^19^

Distractions from frequent guest’s arrival, commitments of house chores and ill mother’s responsibility were other family related problems that were causing the struggling students to lose focus in studies and poor performance in examinations.

### Limitations of the study

We did not analyze the year-wise problems of struggling students due to unavailability of uniform data.

## CONCLUSIONS

Students were struggling more with emotional and family related problems and less with academics related problems. The problems were impacting student’s academics as they were demotivated, lost focus in studies, performed poorly, and failed in examinations. The medical institute had a robust mentoring program however, struggling students need timely identification and more dedicated time to help them manage their problems. Stress relieving activities and counselling sessions may frequently be added as healthy mind and body of the medical students is important for the provision of quality health services to their patients during medical school training and later to the community. Moreover, academically underachieved students should also be given extra care, support, and guidance. Future research should involve multiple institutes to get rich insights into the student’s problems and their experiences with formal mentoring program. Moreover, personal, and professional impact should also be explored along with academic impact.

### Authors’ Contribution

**AA** and **UM** conceived the idea and designed the study.

**AA** collected data. **AA, UM** and **AS** did data analysis and interpretation.

**UM** and **AS** did time to time review and gave feedback to add important intellectual content.

All the authors contributed towards writing the manuscript and approved the final version. All the authors are accountable for integrity of research.
